# Keep the Sprays Away?: Home Pesticides Linked to Childhood Cancers

**Published:** 2007-12

**Authors:** Tina Adler

Previous studies have suggested a link between pesticide use in the home and childhood hematopoietic tumors, the most common type of childhood cancer. A new epidemiologic study of French children diagnosed with leukemia or lymphoma in 2003 or 2004 suggests that a child has about twice the risk of developing acute leukemia (AL) or non-Hodgkin lymphoma (NHL) if his or her mother used insecticides in the home while pregnant **[*EHP* 115:1787–1793; Rudant et al.]**.

The researchers interviewed 1,060 children diagnosed within the prior 6 months and 1,681 control children. When analyzing the data on the children, the team controlled for other factors that may alter a child’s risk of getting cancer, including family cancer history and whether the child was breastfed. The children with cancer were part of the French National Registry of Childhood Blood Malignancies, begun in 1990, which documents all children in the country under age 15 year who have had hematopoietic tumors.

The researchers asked the children’s mothers about their use while pregnant of pesticides in their homes, on pets, and in the garden. They also asked about the father’s use of pesticides while the mother was pregnant and after the child’s birth. Just over 50% of the parents who had a child with AL or NHL had used pesticides at least once during the pregnancy, as did just under 40% of the parents of the control group. Children had 2.1 and 1.8 times the risk of developing AL or NHL, respectively, with maternal use of pesticides during pregnancy.

Mothers’ use of insecticides during pregnancy was significantly associated with childhood AL, NHL, and mixed-cell Hodgkin lymphomas (HLs). Use of pesticides by fathers was also related to AL and NHL. The association was stronger for common B-cell acute lymphoblastic leukemia (ALL) and acute myeloblastic leukemia than for T-cell ALL or mature B-cell ALL. It was also stronger for Burkitt lymphoma than for the other NHLs. Of the HLs, the study linked only the mixed-cell subtype to pesticide use. The strength of the association between pesticide use in the home and cancer did not change as the children grew older.

This is the first study to tease out the different types of hematopoietic cancers as they relate to pesticide use in the home. Other studies have found a link between parents’ occupational exposure to pesticides and childhood cancers, but few of the parents in the French study were exposed to pesticides at work or through farming. Whether a family was rural or urban didn’t alter a child’s risk of developing cancer.

The two types of lymphoma associated with maternal pesticide use during pregnancy have also both been linked to the Epstein-Barr virus, which may suggest a link between pesticide exposure and susceptibility to a viral lymphoma. According to the authors, the consistency of the findings suggests pregnant women may want to avoid pesticide use.

